# Multitasking Na^+^/Taurocholate Cotransporting Polypeptide (NTCP) as a Drug Target for HBV Infection: From Protein Engineering to Drug Discovery

**DOI:** 10.3390/biomedicines10010196

**Published:** 2022-01-17

**Authors:** Dariusz Zakrzewicz, Joachim Geyer

**Affiliations:** Faculty of Veterinary Medicine, Institute of Pharmacology and Toxicology, Biomedical Research Center Seltersberg (BFS), Justus-Liebig-University Giessen, Schubertstrasse 81, 35392 Giessen, Germany; Joachim.M.Geyer@vetmed.uni-giessen.de

**Keywords:** HBV, posttranslational modification, Na^+^/taurocholate cotransporting polypeptide, NTCP, mutation, bile acid transport, virus receptor

## Abstract

Hepatitis B virus (HBV) infections are among the major public health concerns worldwide with more than 250 million of chronically ill individuals. Many of them are additionally infected with the Hepatitis D virus, a satellite virus to HBV. Chronic infection frequently leads to serious liver diseases including cirrhosis and hepatocellular carcinoma, the most common type of liver cancer. Although current antiviral therapies can control HBV replication and slow down disease progress, there is an unmet medical need to identify therapies to cure this chronic infectious disease. Lately, a noteworthy progress in fighting against HBV has been made by identification of the high-affinity hepatic host receptor for HBV and HDV, namely Na^+^/taurocholate cotransporting polypeptide (NTCP, gene symbol *SLC10A1*). Next to its primary function as hepatic uptake transporter for bile acids, NTCP is essential for the cellular entry of HBV and HDV into hepatocytes. Due to this high-ranking discovery, NTCP has become a valuable target for drug development strategies for HBV/HDV-infected patients. In this review, we will focus on a newly predicted three-dimensional NTCP model that was generated using computational approaches and discuss its value in understanding the NTCP’s membrane topology, substrate and virus binding taking place in plasma membranes. We will review existing data on structural, functional, and biological consequences of amino acid residue changes and mutations that lead to loss of NTCP’s transport and virus receptor functions. Finally, we will discuss new directions for future investigations aiming at development of new NTCP-based HBV entry blockers that inhibit HBV tropism in human hepatocytes.

## 1. Introduction

Hepatitis B virus (HBV) infections are one of the major health issues worldwide. The virus causes liver infection, which often leads to acute and chronic hepatic diseases such as fulminant hepatic failure and cirrhosis [1,2]. Moreover, HBV infection is strictly associated with the development and progression of hepatocellular carcinoma, a primary liver cancer and a major cause of death in patients suffering from cirrhosis, an end-stage liver disease [3]. In 2015 the World Health Organization estimated that more than 250 million people around the globe were chronically infected, which consequently resulted in almost 900,000 deaths every year due to HBV-related liver diseases [4]. Since this is the seventh highest cause of worldwide mortality, reducing HBV infections and improving the patients’ treatment is currently aimed in many countries [5,6].

Various vaccines and anti-HBV drugs are available thereby preventing new infections and treating liver diseases in HBV-positive patients, respectively [7,8]. Still, scientific and medical communities have embarked on a concerted journey to identify new antiviral drugs aimed at curing infection [5,7]. Although numerous host-derived proteins have been identified as major contributors to HBV infection thus far, these findings had limited functional outcomes, most likely due to the poorly understood in vivo structure, topology, posttranslational modification, and biomolecule-related interaction of relevant target proteins [5,9,10,11,12]. Moreover, many proteins possess more than one function in the cell. Therefore, therapies aiming to block one particular protein function may often lead to disturbance of another function required for maintaining cell, tissue or organ homeostasis [13,14,15,16]. Thus, many elements of this sensitive protein system must be considered in the discovery of novel drug-based therapies. A deep understanding of three-dimensional structure, topology, cellular expression, and physical interactions of target proteins in this process is essential.

## 2. NTCP: Structure and Transport Activity

### 2.1. NTCP’s Protein Sequence Is Evolutionary Conserved

**Figure 1:**
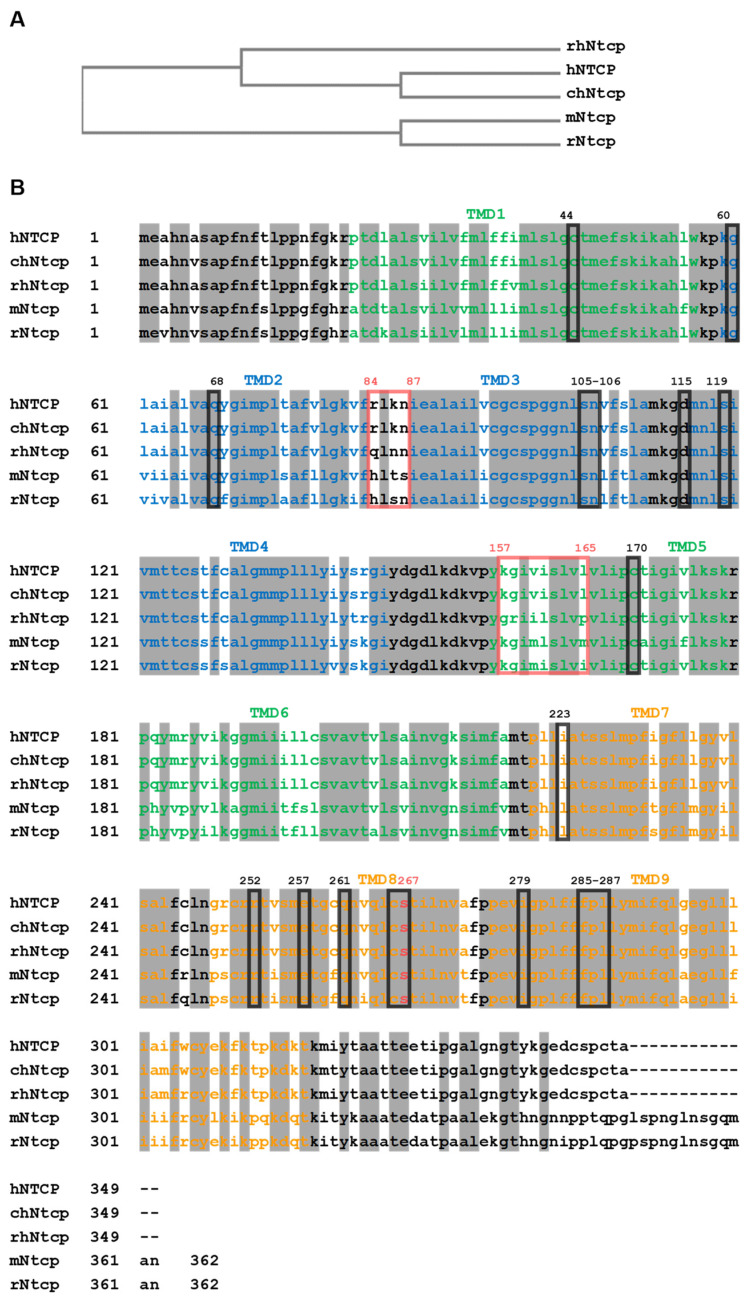
Multiple sequence alignment of NTCP/Ntcps from different species. (**A**) Phylogenetic relationship between human NTCP (hNTCP; Uniprot: Q14973), chimpanzee Ntcp (chNtcp; H2Q8J0), rhesus monkey Ntcp (rhNtcp; F6YRK3), rat Ntcp (rNtcp, Uniprot: P26435) and mouse Ntcp (mNtcp; Uniprot: O08705). (**B**) Deduced amino acid sequences from above-mentioned species were aligned using EBI ClustalW algorithm. Positions of transmembrane domains (TMD) are indicated with the color code also used in Figure 2. Identical amino acids among all species are marked with grey shading. The HBV/HDV preS1-peptide binding motifs of hNTCP _84_RLKN_87_ and _157_KGIVISLVL_165_ are marked with red boxes and amino acids regulating bile acid transport are labeled with black boxes. The highly conserved serine at position 267 that is relevant for bile acid binding and HBV/HDV infection is colored in red.

Human NTCP (hNTCP, gene symbol *SLC10A1*) is the founding member of the Solute Carrier Family SLC10, also known as the sodium-bile acid cotransporter family. Human NTCP consists of 349 amino acids (aa) and so is 13 aa shorter than the mouse (mNtcp) and rat (rNtcp) Ntcp, both with 362 aa [17]. Regardless of the size, these proteins share a high-amino acid sequence identity of 79% (hNTCP vs. mNtcp) and 78% (hNTCP vs. rNtcp) (Figure 1). High structural similarities translate into comparable in vivo properties such as basolateral membrane localization in hepatocytes, Na^+^-dependent transport of bile acids or binding the viral preS1-domain of the HBV/HDV large surface proteins [9,13,18,19,20]. Moreover, a recent study focusing on functional and pharmacological comparison of hNTCP and mNtcp found strong correlation between the transport kinetics and inhibition pattern among both proteins [17]. This strongly suggests that most of the data generated on rodent Ntcps might also be translated to research on hNTCP, thereby supporting current NTCP-based drug discoveries. However, despite the fact that the interaction of drugs and pesticides with hNTCP and mNtcp is comparable, specific compounds such as glibenclamide, rosuvastatin, benzbromarone and rifampicin showed clear species differences, which have to be considered when pharmacokinetic data are compared between mice and humans [17].

### 2.2. Prediction of Human NTCP Structure: Homology and Computational Approaches

**Figure 2:**
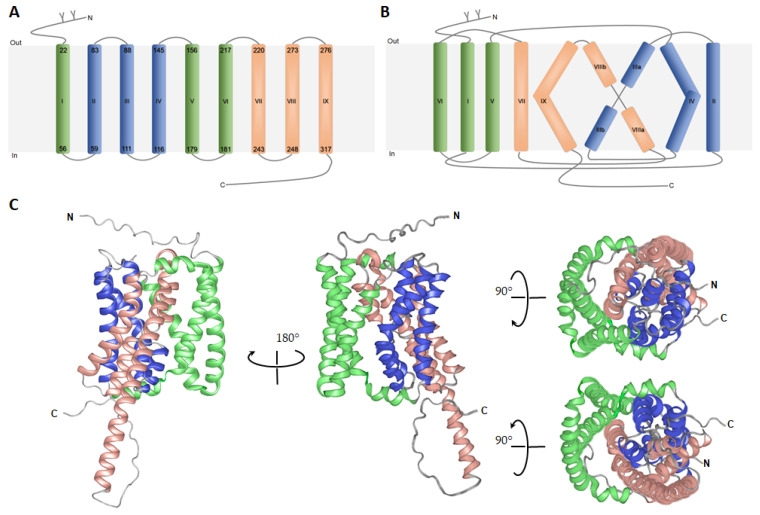
Three-dimensional model of human NTCP predicted using AlphaFold. (**A**) Schematic representation of nine transmembrane domains (TMDs I-IX) of human NTCP with indicated aa positions of the α-helices (Arabic numbers). Transmembrane domains are marked (Greek letters) and colored: I, IV and V (green, panel domain); II, III, IV (blue, core domain); and VII, VIII and IX (orange, core domain). N-terminal glycosylation of the N5 and N11 are demonstrated as “Y”. (**B**) Proposed membrane topology of human NTCP based on AlphaFold prediction (AF-Q14973-F1-model_v1). (**C**) Backbone structure of human NTCP protein, where α-helices are represented by coiled ribbons, and protein loops are shown as thin lines. Positions of N- and C-termini are labeled. Two identical structures are related by a 90-degree rotation. The model was visualized by the Protean 3D DNASTAR Software.

Apical sodium-dependent bile acid transporter (ASBT, gene symbol *SLC10A2*), also known as ileal bile acid transporter (IBAT), is the second relevant bile acid transporter of the SLC10 family expressed in the apical domain of ileal enterocytes [21]. In the past two ASBT-related crystal structures were obtained from *Neisseria meningitidis* (Asbt_Nm_) [22,23] and from *Yersinia frederiksenii* (Asbt_Yf_) [24]. They both share approximately 25% protein sequence homology with ASBT as well as with NTCP [22,24,25]. Based on these structures, numerous homology models have been generated for hASBT and hNTCP, giving a first impression about their membrane topologies, conformation, and underlying mechanisms of the bile acid transport process [24,26,27]. During the last years, bioinformatics and computational tools for protein structure prediction have made great progress and are being increasingly used to analyze and to make predictions about the structure of proteins and protein complexes [28,29]. Lately, a new three-dimensional structure of NTCP protein has been generated with the highly accurate protein structure prediction platform called AlphaFold [30,31]. It is a novel machine learning approach that incorporates physical and biological knowledge about protein structure, leveraging multisequence alignments, into design of the deep learning algorithm [30]. This progress resulted in structure predictions for many integral membrane proteins such as hNTCP (Sequence Nr.: AF-Q14973-F1) allowing deeper understanding of protein folding, as well as the role of particular amino acids for molecule function and interactions (Figure 2) [30,32].

As the structure generally dictates protein function, this AlphaFold-based model may not only help to study NTCP‘s transporter activity, ligand/receptor interaction or oligomerization, but might also provide valuable information on dynamic interactions in living cells required for NTCP-dependent HBV/HDV entry into human hepatocytes. Over the last years, NTCP topology has been widely discussed and several structures of NTCP have been proposed [24,26,27]. According to most recent studies (based on the bacterial Asbt crystal structures and the AlphaFold prediction), human NTCP possess nine transmembrane (TMD) helices [18,23] with an extracellular glycosylated amino terminus and a C-terminal arm localized intracellularly (Figure 2A) [18,23,33,34]. These transmembrane helices are arranged into a “core” domain composed of TMDs II, III, IV, VII, VIII, and IX, and a “panel” domain comprised of TMDs I, V and VI. Both domains seem to flank a substrate binding crevice (Figure 2B,C). A homology model of hNTCP, based on the crystal structure of bacterial Asbt from *Yersinia frederiksenii*, supposed that bile salt transport is coupled to a conformational change in the transporter protein that is promoted by the rotation of TMD II [22]. Another model of NTCP based on the Asbt crystal structure from *Neisseria meningitidis*, predicted the surface regions, which are essential for HBV/HDV binding to NTCP and for infection of NTCP-expressing hepatoma cells [22]. These two NTCP sequences _157_KGIVISLVL_165_ and _84_RLKN_87_ (detailed discussed in the next chapter) are localized in TMD V and extracellular loop I, respectively [22]. Despite the immense value of the above-mentioned discoveries, all these data still require further experimental verification using virus/NTCP complexes subjected to X-ray crystallography, NMR spectroscopy or electron microscopy analyses.

### 2.3. NTCP Primary Function: Bile Acid Uptake

NTCP is a membrane-localized protein, which is produced in the liver, where its unique expression defines its primary function [18,19]. In hepatocytes, NTCP acts as a transporter that is responsible for the sodium-dependent uptake of bile acids from the portal blood into hepatocytes through their sinusoidal/basolateral plasma membrane [18,19]. The list of bile acid molecules that are transported by NTCP is long and includes: cholic acid, chenodeoxycholic acid, deoxycholic acid, ursodeoxycholic acid, sarcosine cholic acid, glycocholic acid, glycochenodeoxycholic acid, glycodeoxycholic acid, glycoursodeoxycholic acid, taurocholic acid (TC), taurochenodeoxycholic acid (TCDC), taurodeoxycholic acid, tauroursodeoxycholic acid (TDC) and taurolithocholic acid (TLC) [27]. It seems that NTCP transports all major physiologically relevant glycine and taurine-conjugated bile acids, although transport of the sulfated steroids estrone-3-sulfate, DHEAS and pregnenolone sulfate [35] as well as thyroid hormones by NTCP have also been reported [36,37].

Due to the high NTCP expression in the sinusoidal membrane of hepatocytes and its extreme high affinity to conjugated bile acids, NTCP efficiently extracts them from the portal blood and thereby maintains enterohepatic circulation of bile acids and keeps plasma concentrations at minimum [18,19,38]. Recently, it was proposed that a large substrate/inhibitor entry zone exists in the outward oriented space between the “core” and the “panel” domains of NTCP that is characterized by multiple interaction domains for different kind of substrates such as TLC, TC, DHEAS, and different classes of inhibitors. Hence, TLC may have a potent inhibitory effect on the TC and DHEAS transport function of NTCP [39,40]. In addition, rapid accumulation of TLC mediates long-lasting *trans*-inhibition of the transporter and receptor functions of NTCP, most likely via an intracellular TLC-binding domain of NTCP [40]. Noteworthy, as a bile acid transporter, NTCP may also be responsible for the uptake of drugs and other xenobiotics, for instance rosuvastatin, fluvastatin, atorvastatin as well as pitavastatin, irbesartan, ezetimibe and losartan, thereby influencing bile acid circulation [41,42,43,44,45].

Numerous inhibitors can block or significantly influence the NTCP-mediated bile acid transport through the hepatic plasma membrane. The most characterized are cyclosporine A, propranolol, furosemide, ketoconazole, rifamycin, glibenclamide, ritonavir, bosentan, efavirenz, saquinavir, and gemfibrozil [21,46]. By inhibiting NTCP-mediated bile acid influx into hepatocytes, these drugs can cause bile acid elevation in the blood by the mechanism involving a drug-bile acid interaction at NTCP’s substrate/inhibitor binding site. In a similar manner, NTCP deficiency can increase plasma bile acid levels. Indeed, extremely high concentration of bile salts was measured in NTCP-deficient individuals, thereby manifesting in conjugated hypercholanemia with no apparent long-term clinical consequences [20,47]. Although the elevated bile acids in the blood circulation are well-tolerated, the metabolic effects related to their relatively high levels, for instance changes in CYP3A activity or release of glucagon-like 1 (GLP-1), have been reported [48,49,50]. This could be due to the regulation and redistribution of bile acids as signaling molecules through other transporters such as ASBT, organic anion transporting polypeptides (OATPs), or canalicular efflux transporters such as MRP2, MRP3 and MRP4 [51]. The long-term effects of elevated bile acids in human blood will require additional complex studies and critical evaluation.

Considering all potential substrates and inhibitors of NTCP, regulation of NTCP transporter activity is a very dynamic and sensitive process and it may significantly be influenced by various exogenous and endogenous compounds in vivo leading to unpredictable long-lasting consequences. Therefore, designed drugs aiming at blocking other NTCP functions such as HBV receptor interaction shall not impact on or interfere with NTCP-driven bile acid uptake into hepatocytes.

## 3. Protein Engineering as a Valuable Approach to Study NTCP Functions

An important prerequisite for drug development is a deep understanding of tertiary/quaternary structures of target proteins and their undergoing conformational transitions upon ligand binding. Nuclear magnetic resonance (NMR) spectroscopy and X-ray crystallography are still the main approaches to determine unique macromolecule structures and to detect their dynamics in solution [52,53]. More recently, cryoelectron microscopy (cryo-EM) has gained significant improvements regarding resolution and provides several advantages: (I) only a small amount of protein is required, (II) there is no need for protein crystal formation, and, (III) most importantly, proteins can be studied in their natural, hydrated state, in a biologically relevant environment [54,55,56]. However, all above-mentioned methods require time-consuming and lab-intense purification of stable, correctly folded and functionally active proteins, which, in the case of membrane receptors/transporters such as NTCP, is very challenging. Apart from the predicted models based on the protein structure homology or highly accurate computational approaches such as AlphaFold [22,23,24,30], the exact structure of human NTCP has thus far not been reported. Hence, instead of analyzing hypothetical 3-D protein models, introducing missense mutations into target protein is an alternative option for studying the role of individual amino acids in protein folding, stability, and molecular function [33,57,58,59,60,61]. This mimics conformational changes upon substrate uptake and ligand binding [26,62]. Site-directed mutagenesis of one or more amino acids followed by functional analyses in vivo may answer the question whether or not and to what extent a certain amino acid residue participates in protein interactions [63]. Although protein engineering may increase the misfolding/unfolding propensity of proteins in vitro [33,60,61], when properly controlled, it still provides inestimable information on protein structure determination, topology, protein–protein interactions, as well as substrate/ligand–protein synergy.

Over the last decade, the NTCP/Ntcp protein underwent detailed characterization using experimental approaches involving protein engineering of human NTCP and rodent Ntcps in vitro. Single mutations, deletions, and fragment replacements within the NTCP/Ntcp sequence have identified numerous regions and residues that define the protein structure, topology and its functionality. As depicted in Table 1, those studies were mainly focused on: (i) Na^+^-dependent bile acid uptake and substrate specificity [20,33,40,46,48,64,65,66,67,68], (ii) HBV/HDV receptor function [9,13,33,34,40,63,68,69,70,71,72], (iii) cellular localization and protein trafficking [33,39,40,64,65,73,74], (iv) posttranslational modifications [33,34,39], (v) oligomerization [63,71,75,76], and (vi) the presence of conserved motifs or functional domains [13,39,62,64,67,71,77].

**Figure 3:**
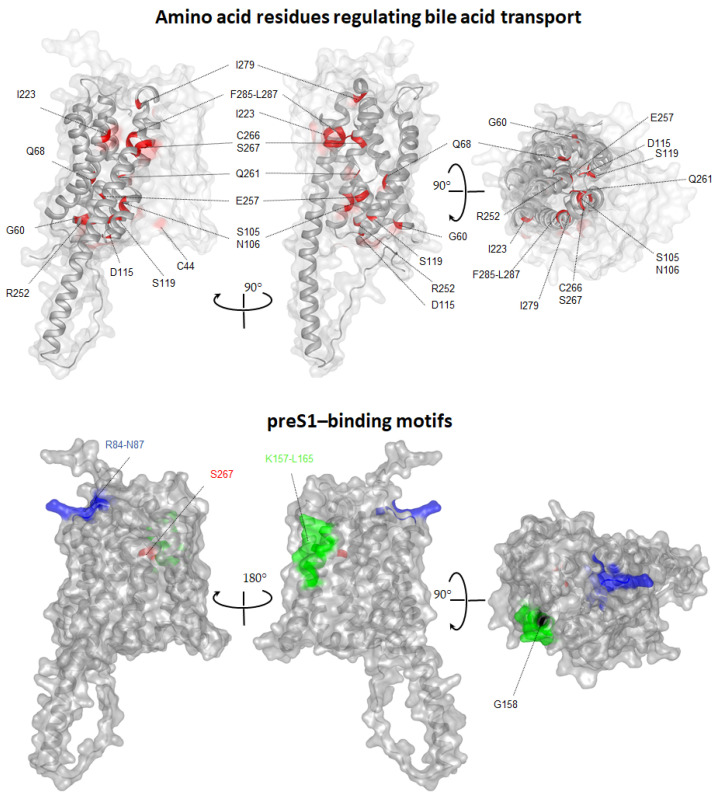
NTCP regions and amino acids essential for bile acid transport and HBV/HDV binding. The AlphaFold model of human NTCP (AF-Q14973-F1) was visualized with Protean 3D DNASTAR Software. (Top panel) To better visualize amino acids regulating bile acid transport, the NTCP “panel” domain (TMDs I, V and VI) was made transparent. Positions of core-localized amino acids, namely C44, G60, Q68, S105, N106, D115, S119, C170, I223, R252, E257, Q261, C266, S267, I279, F285, P286 and L287 are marked and colored in red. Two identical structures are related by a 90-degree rotation. (Lower panel) Transparent surface presentation of NTCP. Positions of amino acids involved in preS1-binding activity are labeled with colors (_157_KGIVISLVL_165_ (green), 158G (black), _84_RLKN_87_ (blue), 267S (red)) (top panel)**.** Two identical structures are related by a 180 and 90-degree rotation.

Until now, more than seventy aa from the entire 349 aa hNTCP sequence have been subjected to mutations, which resulted in approximately 120 NTCP/Ntcp mutants generated (Table 1). The largest number of them examined residues that participate in bile acid transport. According to previous reports, amino acids located at the N-terminal part of NTCP/Ntcp, such as D24, C44, G60, A64, Q68, S105, N106, D115, S119 have been reported to markedly regulate the bile acid transport function [66,71,75,78]. Moreover, strong evidence suggests that transmembrane domain VIII, which is localized in the “core” domain, is essentially involved in the bile acid transport activity. This TMD contains many critical aa, namely C250, R252, E257, C260, Q261, C266 and S267 [20,26,62,66,67,78,79]. S267F and R252H are natural NTCP variants, both causing an NTCP loss-of-function phenotype. In addition, S267F has protective effects against HBV and HDV infection and reduced the risk of developing liver cirrhosis and hepatocellular carcinoma [20,26,68]. This S267-dependent antiviral activity may interfere with the transport of physiological substrates of NTCP, suggesting a common binding pocket near S267 for both the substrate and the virus-derived preS1-peptide [22,26]. To support the observation that the S267 residue-containing domain displays such multifunctional characteristics, it has been reported that the residues N262, Q293 and L294 exhibited strong bile acid-binding [78]. Furthermore, E257 and Q261, but also Q68, S105 and N106, have been shown to be presumably involved in binding of the cosubstrate Na^2+^ and, in addition, in the regulation of viral infection, suggesting that residues involved in substrate transportation may also be utilized upon HBV infection [77,78]. Altogether these studies suggest that a multifaceted S267-containg-fragment (C250-L294) plays a pivotal role in maintaining NTCP functionality, which includes Na^+^-binding, substrate transport, and HBV internalization (Table 1). The most recent 3-D model of hNTCP confirmed experimental observations and demonstrated the presence of C44, G60, Q68, S105, N106, D115, S119, C170, I223, R252, E257, Q261, C266, S267, I279, F285, P286 and L287 residues in the protein “core” domain of NTCP. More importantly, all these amino acids together build a kind of substrate “tunnel”, allowing the bile acids to pass through the protein using space between the “panel” and “core” domains of NTCP (Figure 3, upper panel).

**Table 1:** Mutations in human NTCP and rodent Ntcps that were reported to affect folding, structure, expression, and functions.

Mutation	Functional Consequences	Ref.
**N5Q**	- no effect on HBV infection [33,34] - no effect on TC uptake [33] - blocked partially NTCP glycosylation [33,34] - no effect on NTCP plasma membrane expression [33,34]	[33,34]
**N5A**	- blocked partially N-glycosylation - no effect on HBV infection - no effect on plasma membrane expression	[34]
**N5Q/N11Q**	- inhibited HBV infection (conflicting data) [33] - reduced TC uptake [33] - completely abolished N-glycosylation [33] - no plasma membrane expression, protein rapidly undergoes endocytosis to be degraded in lysosomes [33] - no effect on HBV infection (conflicting data) [34] - no effect on plasma membrane expression [34]	[33,34]
**N5A/N11A**	- completely abolished NTCP N-glycosylation - no effect on HBV infection - no effect on NTCP plasma membrane expression	[34]
**N11Q**	- no effect on HBV infection [33,34] - no effect on TC uptake [33] - partially blocked NTCP N-glycosylation [33,34] - no effect on NTCP plasma membrane expression [33,34]	[33,34]
**N11A**	- blocked partially glycosylation - no effect on HBV infection - no effect on plasma membrane expression	[34]
**D24N**	- disturbed NTCP membrane localization (amino acid residue essential for appropriate protein sorting) - inhibited TC transport	[66]
**V29I/I38V**	- no effect on NTCP surface expression - no effect on preS1 peptide binding - no effect on HBV infection	[13]
**C44A**	- no effect on transport	[66]
**C44W**	- reduced slightly TC uptake	[66]
**C44T**	- reduced TC uptake	[66]
**E47Q**	- no effect on TC transport	[66]
**G60L**	- disturbed protein folding and sorting of NTCP, and so indirectly affected protein glycosylation, homodimerization, and bile acid transport of NTCP - reduced HBV/HDV receptor function - is part of the G60XXXA64 dimerization motif	[71,75]
**A64L**	- disturbed proper folding and sorting of NTCP - indirectly affected protein glycosylation, homodimerization, and bile acid transport of NTCP - reduced HBV/HDV receptor function - is part of the G60XXXA64 dimerization motif	[71,75]
**Q68A**	- inhibited TC uptake - reduced HBV and HDV infection - no effect on NTCP surface expression	[78]
**K81-S119** (Replacement)	inhibited HBV infection	[9]
**84RLKN87** (Replacement)	- reduced HBV infection - diminished preS1-peptide binding	[9,77,80]
**R84H**	- no effect on NTCP membrane localization - no effect on TC transport	[77]
**K86T**	- no effect on membrane localization - no effect on TC transport	[77,80]
**R84Q/K86N**	- no effect on surface expression - no effect on preS1 peptide binding - no effect on HBV infection	[13]
**N87S**	- no effect on membrane localization - no effect on TC transport	[77]
**E89Q**	- no effect on TC transport	[66]
**C96A**	- reduced Na^+^-dependent TC transport	[66]
**C96W**	- reduced TC transport	[66]
**C98A**	- no effect on TC transport	[66]
**C98W**	- inhibited TC transport	[66]
**S105A/** **N106A**	- inhibited TC uptake - no effect on NTCP total protein expression and surface expression - no effect on preS1 peptide binding - inhibited HBV, but not HDV infection	[78]
**D115N**	- inhibited TC transport	[66]
**S119A**	- reduced TC uptake	[78]
**120I-S178** (replacement)	- no effect on HBV infection	[9]
**T123A**	- reduced NTCP total and surface expression	[78]
**C125A**	- no effect on TC transport	[66]
**C125del**	- inhibited TC transport	[66]
**L136A/** **L137A**	- no effect on NTCP total and membrane expression, - is part of the di-leucine motif L136L137	[73]
**I140L/S142T**	- no effect on surface expression - no effect on preS1 peptide binding - no effect on HBV infection	[13]
**G144A/G148A**	- reduced pres1 peptide binding - diminished interaction with EGFR - is part of the G144XXXG148 motif	[70]
**D147N**	- no effect on TC transport	[66]
**157KGIVISLVL165** (Replacement)	- inhibited HBV infection - inhibited preS1-peptide binding - no effect on surface expression	[9,13]
**G158R/D/N/V/S**	- no effect on transport activity - diminished HBV receptor function: reduced preS1-petide binding and inhibited HBV infection	[62]
**S162A/** **L163A**	- no effect on TC uptake	[78]
**C170A**	- no effect on TC transport	[66]
**C170W**	- inhibited TC transport	[66]
**S213A**	- no effect on PKC-mediated endocytosis	[73]
**T219A**	- no effect on PKC-mediated endocytosis	[73]
**L222A**	- disturbed NTCP membrane expression and induced clathrin-mediated endocytosis - no effect on NTCP total expression - accumulated an immature core-glycosylated NTCP and reduced a mature complex-glycosylated NTCP - is part of the di-Leucine motif L222L223 in rat Ntcp	[67,73]
**I223T**	- reduced TC, cholate and estrone-3-sulfate uptake - reduced NTCP membrane expression - no effect on NTCP total expression - increased intracellular retention	[67]
**T225A**	- inhibited PKC-mediated endocytosis - is part of the internalization motif	[73]
**T225A/S226A**	- no effect on PKC-mediated endocytosis	[73]
**S226A**	- inhibited PKC-mediated endocytosis - is part of the internalization motif	[73]
**S227A**	- no effect on PKC-mediated endocytosis	[73]
**G233L**	- disturbed proper folding and sorting of NTCP, and so - indirectly affected protein glycosylation, homodimerization, and bile acid transport of NTCP - reduced HBV/HDV receptor function - is part of the G233XXXG237 motif	[71,75]
**G237L**	- disturbed proper folding and sorting of NTCP, and so - indirectly affected protein glycosylation, homodimerization, and bile acid transport of NTCP - reduced HBV/HDV receptor function - is part of the G233XXXG237 motif	[71,75]
**C250A**	- no effect on TC transport	[66]
**C250Del**	- inhibited TC transport	[66]
**R252H**	- inhibited TC uptake - reduced NTCP glycosylation - no effect on NTCP core-glycosylated NTCP expression, - disturbed NTCP plasma membrane expression - increased level of serum bile acids (patient data) - no severe liver dysfunctions (patient data)	[20]
**E257Q**	- inhibited TC transport	[66]
**E257A**	- inhibited TC uptake - no effect on NTCP total and surface expression - inhibited preS1 peptide binding - inhibited HBV and reduced HDV infection	[78]
**T258A**	- no effect on TC uptake	[78]
**C260A**	- reduced TC uptake	[78]
**Q261A**	- inhibited TC uptake - no effect on NTCP total and surface expression - reduced slightly preS1 peptide binding - inhibited HBV infection, but no effect on HDV infection	[78]
**N262A**	- inhibited TC uptake [72] - no effect on NTCP total and surface expression - inhibited preS1 peptide binding - inhibited HBV and HDV infection	[78]
**C266A**	- reduced TC transport	[66]
**C266Del**	- inhibited TC transport	[66]
**S267F**	- no effect on TC uptake [78] - inhibited preS1 peptide binding [78] - inhibited HBV and HDV infection [78] - reduced bile acid transport, but not rosuvastatin and estrone-3-sulfate (patient data) [67], - diverse effect on disease progression during HBV infection (patient data) [68] - prone to Vitamin D deficiency (patient data) [81]	[62,67,68,78,79]
**S267/X** (X represents 19 aa used in the study)	- varied TC uptake, estrone-3-sulfate and rosuvastatin, depending on point mutation (X) - altered NTCP surface expression in various S267 mutants	[26]
**F274A**	- loss of HBV susceptibility - disrupted NTCP oligomerization - inhibited HBV internalization	[63]
**E277Q**	- no effect on TC uptake	[66]
**I279T**	- reduced TC, cholate and estrone sulfate uptake - no effect on NTCP membrane and total expression	[67]
**F285A/P286A/L287A**	- inhibited TC uptake - disturbed NTCP total and cell expression	[78]
**Q293A/L294A**	- reduced TC uptake - no effect on NTCP total and surface expression - no effect on preS1 peptide binding - inhibited HBV, but not HDV infection	[78]
**I303M/R305W**	- no effect on surface expression - no effect on preS1 peptide binding - no effect on HBV infection	[13]
**C306A**	- no effect on TC transport	[66]
**C306W**	- no effect on TC transport	[66]
**C306Del**	- inhibited TC transport	[66]
**Y307E/K/I** **Y307Stop**	- disturbed NTCP membrane trafficking (C-terminal membrane localization sequence)	[64,65]
**K314E**	- reduced TC, cholate and estrone3-sulfate - no effect on NTCP membrane and total expression	[67]
**T317A**	- no effect on TC transport	[66]
**T317Y**	- no effect on TC transport	[66]
**T320A**	- no effect on TC transport	[66]
**T320Y**	- no effect on TC transport	[66]
**Y321K/A/A**	- disturbed NTCP membrane trafficking - (C-terminal membrane localization sequence)	[64,65,75]

Extensive in vitro studies involving site-directed nonsense and missense mutations revealed that the C-terminal part of NTCP is also relevant for bile acid transport activity. In line with this, deletion of the C306 (but not replacement of C306 with alanine or tryptophan) and substitution of K314 with glutamic acid have demonstrated a vast impact on the NTCP-mediated TC uptake by hepatocytes [66,67]. Moreover, the C-terminal part of NTCP contains a membrane localization sequence, which seems to be localized within the last 40–100 aa residues [64,65,75]. Experiments involving the stop codon mutation at C250 and Y307 positions as well as aa substitutions of Y307 and Y312 consequently led to dramatic reduction of membrane expression and increased intracellular retention of NTCP [64,65,75]. Hence, since TC uptake by hepatocytes is positively correlated with NTCP surface expression, it is not surprising that modifications of this regions are responsible for (i) NTCP membrane trafficking (residues C250, Y307, Y312); (ii) PKC-mediated NTCP endocytosis (residues T225, S226); (iii) protein sorting and clathrin-mediated cellular trafficking (residues D24, L222, I223); (iv), protein oligomerization/dimerization/homodimerization (G60XXXA64, G144XXXG148, G233XXXG237 motifs), which also indirectly influenced NTCP-dependent bile acid metabolism.

The amino-terminus of NTCP undergoes posttranslational N-glycosylation, which plays a fundamental role in NTCP biology [19,33]. Although glycosylation of N5 and N11 residues of NTCP is not a matter of debate, the functional consequences of N-terminal asparagine modifications is controversial [33,34]. It has been evidenced that loss of one N-terminal asparagine (N5A, N5Q, N11A, or N11Q) had no effect on NTCP surface expression and HBV infection [33,34]. Moreover, the single mutation of N5 or N11 to alanine or glutamine did not significantly change TCA transport activity of the protein. However, NTCP double mutants N5A/N11A and N5Q/N11Q lacking both N-glycosyl moieties were unable to transport bile acids. This suggests that only one glycan residue of NTCP (N5 or N11) is required for bile acid uptake by hepatocytes [33]. Regarding HBV infection, the role of N-glycosylated asparagines of NTCP in this process is still unclear. Mutations of both (N5Q/N11Q or N5A/N11A), from one hand, drastically reduced plasma membrane abundancy of NTCP and, thus, markedly diminished NTCP-driven HBV entry into hepatocytes [33], but, on another hand, Lee et al. proposed that glycosylation of NTCP is not required for HBV infection, as the virus could still enter hepatocytes expressing the glycosylation-deficient form of NTCP [34]. Hence, the impact of N-terminal glycosylation of NTCP on HBV entry into hepatocytes remains questionable and it needs further investigation.

Comprehensive mutational analysis of NTCP protein had a great value in defining two protein regions, namely _84_RLKN_87_ and _157_KGIVISLVL_165_ (Table 1), which interact with the preS1 domain of the large surface protein of HBV, strongly supporting the notion that NTCP is the functional HBV receptor [9,13,77,78]. Furthermore, NTCP mutants of S267 and G158 help to decipher the fundamental role of these two residues in the NTCP-mediated virus tropism. This aspect will be discussed in the next paragraph.

Although highly informative, previously published results from NTCP protein engineering followed by the experimental characterization of generated mutants have noticeable limitations. Thus, the conclusions from these observations should be drawn with precaution. Apparently, many studies did not evaluate whether the surface localization of generated mutants was unaffected. Moreover, only some investigators considered protein stability as a key parameter, which appears to be essential for protein –protein interaction, dimerization and cellular trafficking of membrane-expressed proteins [26,33]. Changes in stability due to aggregation, degradation, chemical or physical instability can alter protein folding and structure, which are essential features required for natural protein activity, protein–protein interactions, protein oligomerization/homodimerization and cellular expression [9,13,29,60,61,77,78]. Therefore, early assessment of stability with prediction of stability testing studies as well as determination of the proper folding and surface localization of NTCP mutants are fundamental to draw appropriate conclusions from mutation studies.

## 4. NTCP: A Promising Target for Drug Discovery against HBV/HDV Infection

HBV comprises four open reading frames coding for hepatitis B (HB) surface antigen, HBe antigen/HB core antigen, polymerase, and HBV X protein. HB surface antigen consists of three species: small (S-), middle (M-), and large (L-)HB surface proteins [2,82]. The middle and large surface proteins contain so-called preS1 and preS2 regions, which mediate diverse functions in nucleocapsid binding and receptor recognition [10]. HBV entry into hepatocytes is a complex process consisting of different steps, in which several viral and host macromolecules are involved [10,83]. First, the virus connects with the host via low-affinity binding to heparan sulfate proteoglycans on hepatocytes. This process is believed to be mediated by the S-surface protein and the preS1 region of the large L-HB protein. Afterwards, the virus interacts with host-surface protein receptors in order to internalize using the clathrin-mediated endocytosis pathway [25,82,84]. In 2012, NTCP was identified as the high-affinity hepatic receptor for both HBV and HDV [9,13] and this discovery has provided a valuable information for the development of a novel class of HBV/HDV entry inhibitors acting via NTCP inhibition [46,85,86].

The first step in HBV uptake and infection is the high-affinity interaction, which occurs between the myristoylated preS1-lipopeptide comprising the N-terminal amino acids 2–48 (myr-preS1) of the L-HB virus surface protein and hNTCP [74]. The hNTCP protein engineering followed by extensive mapping studies identified fragments of hNTCP that play an essential role in this process (Table 1). For instance, the hNTCP motif _84_RLKN_87_, which differs between hNTCP and mNtcp is fundamental for virus entry and productive HBV infection [9,13,80]. According to the hNTCP homology models based on the crystal structure of Asbt from *Neisseria meningitides* as well as to the AlphaFold NTCP structure, this motif is localized in the extracellular loop between TMD II and TMD III [23,24] and, therefore, is exposed on the protein surface allowing free access of potential interacting viral particles (Figure 3, lower panel). Mutations of particular aa within this motif, such as R84, K86T, R84Q/K86N and N87, had no significant effect on NTCP-driven TC uptake suggesting that this NTCP region is restricted to HBV receptor activity, and it does not play a direct role in the bile acid transport function of NTCP (Table 1).

Mutation studies also identified region _157_KGIVISLVL_165_ as a critical binding motif necessary for interaction between the preS1 domain and hNTCP [9,77]. This motif seems to be relatively conserved between different species. However, a recent study by Müller et al. identified aa G158 of hNTCP as the most relevant amino acid within this motif [62]. G158R mutation toward the respective amino acid of the HBV/HDV insensitive rhesus monkey Ntcp (being R158) completely abolished preS1 peptide binding to NTCP and in vitro HBV/HDV infection of hepatoma cells expressing an G158R NTCP variant. Conversely, introduction of amino acid G158 into the rhesus monkey Ntcp by R158G mutation made this monkey Ntcp susceptible for preS1 peptide binding and at least partially recovered in vitro HBV/HDV infection [62] (Table 1). Therefore, the residue 158 of NTCP was sufficient to discriminate between the HBV/HDV susceptible group of humans and great apes (all bearing G158) and the nonsusceptible group of Old World monkeys such as macaques and baboons (all bearing 158R) [62,87]. Based on the AlphaFold NTCP structure, amino acid 158 is exposed to the extracellularly accessible domain of NTCP, directly preceding membrane insertion of TMD V [62] (Figure 2 and Figure 3). Since it was suggested that bile salt transport is facilitated by movement of the more flexible “panel” domain (TMD I, V, VI) against the more rigid “core” domain (TMD II–IV and VII–IX), the amino acid 158 lays near the proposed entry site for bile salts. This would structurally explain why bile acids as substrates of NTCP could efficiently block myr-preS1 peptide binding to NTCP [62,69].

Another NTCP amino acid residue, which is relevant for HBV/HDV infection, is a serine at aa position 267. The genetic missense rs2296651 variant (c.800C > T, S267F) has been identified in East Asian, but not in African and European patients, and it has been associated with a reduced risk for HBV and HDV infection as well as a slowing disease progression in chronically ill HBV patients [68,79]. In vitro generated mutants, where S267 was substituted with various other amino acids and, in addition, genetically edited hepatic cells expressing the NTCP-S267F variant were resistant to HBV infection, clearly demonstrating the importance of the serine residue in NTCP-driven preS1 peptide binding and HBV internalization [72,78]. Noteworthy, the genetically modified NTCP S267F variant was characterized by a similar TC uptake activity as wild-type NTCP [78]. In contrast, in vivo data from patients carrying the missense variant S267F demonstrated reduced bile acid transport, but nearly intact rosuvastatin and estrone-3-sulfate transport, concomitantly with diverse effects on liver disease progression during HBV infection [79]. More detailed in vitro analysis using series of S267 mutants then revealed that transport of TC, estrone-3-sulfate and rosuvastatin, thus substrate specificity, is depended on the exact structure, physical interaction and chemical characteristics at exactly this aa position [26].

According to the AlphaFold NTCP structure, S267 is located in transmembrane domain TMD VIII, and so near the proposed substrate-binding cavity [22]. Hence, substitutions of the S267 with various amino acids may directly influence substrate transport [26]. Despite protective effects of the S267F variant against HBV and HDV infection, its interference with the bile acid transport might exclude this amino acid as drug target position, at least when sustained bile acid transport activity of NTCP is intended. Nevertheless, except this amino acid residue, all other NTCP motifs involved in NTCP/receptor interaction are exposed on the protein surface. The motif _84_RLKN_87_, located in the extracellular loop connecting TMD II and III, and _157_KGIVISLVL_165_, representing the outer part of TMD V, are more or less exposed to the outer surface of the NTCP protein, supporting their role as preS1 peptide receptor binding motifs (Figure 2 and Figure 3). It is imaginable that such conformation allows for simultaneous and collaborative binding of both NTCP regions to the preS1 domain of the L-HB surface protein. Consequently, both regions are very promising targets for drug discovery aiming at blocking HBV/NTCP interaction.

The discovery of NTCP as the high-affinity functional HBV/HDV receptor opened an ongoing search for new treatments of chronically infected HBV patients. The first identified compounds against HBV infection were major physiological substrates of NTCP, since they compete with the virus for NTCP-interacting domains [43,86]. Hence, TC, TDC, TCDC and their derivatives were discovered to be very potent physiological competitors of HBV/HDV binding to NTCP [43].

Among thirty-one FDA-approved compounds that are able to inhibit NTCP-dependent bile acid uptake are drugs such as irbesartan, ezetimibe, ritonavir, propranolol, progesterone, bosentan and the immunosuppressant cyclosporine A (CsA) [88,89]. CsA may inhibit HBV infection in NTCP-dependent manner by interfering with the preS1 peptide and NTCP interaction with IC_50_ 0.31–1.2 µM [90]. In comparison, the myristoylated preS1 peptide itself is able to block HBV infection with high efficacy at an IC_50_ of 80 pM, thereby giving prerequisite for the development of a therapeutic drug called Myrcludex B [91]. Regardless of its antiviral properties, it also inhibits NTCP-mediated bile acid uptake in vivo [48,50], causing asymptomatic, but distinct rise in plasma bile acid concentrations in healthy human [50]. It is worth mentioning that Myrcludex B has an IC_50_ value for bile acid transport of 52.5 nM in primary human hepatocytes, but blocks HBV infection at far lower concentration (IC_50_ value for HBV infection of 80 pM) [92].Hence, this synthetic lipopeptide consisting of aa 2–48 of the preS1 region has already proven the beneficial effect in fighting infection in phase IIb/IIa clinical trials demonstrating significant reduction of HDV RNA levels in patients chronically coinfected with HBV and HDV after 6 months of monotreatment [93]. Additional clinical trial phases confirmed the effectiveness of Myrcludex B and resulted in its approval for the treatment of a chronic HDV infection under the name Bulevirtide (Hepcludex^®^) [19].

Next to Myrcludex B, which represents a peptide-based approach, there are large numbers of neutralizing antibodies, which target the preS1-region of the virus. For instance, MA18/7, KR 127, BX-182 and 2H5-A14 antibodies have been reported to inhibit HBV and HBV infection in primary hepatocytes and animal models of viral infection [86,94]. In contrast to antibodies that block the virus binding to the cell, there is a body of evidence that neutralizing antibodies directed against NTCP epitopes may block the process of virus internalization. Lately, a monoclonal antibody 18D1 has been reported to specifically interacts with the _84_RLKN_87_ motif of NTCP, and, consequently, had a neutralizing effect against HDV infection in vitro and partial inhibitory effects in an in vivo mouse model [80].

Every year, newly discovered and tested compounds appear on the list of approved drugs for the treatment of HBV and HDV infections. Due to their limited effectiveness and side effects, there is still an unmet medical need for the further development of novel, more specific NTCP-based inhibitors that would cure chronically infected patients. Well-established computational approaches such as pharmacophore methods, quantitative structure-activity relationship (QSAR), free-energy calculations, or docking studies that identify small molecule inhibitors of NTCP are powerful tools for drug design. Such virtual screenings have already been successfully performed bringing new insights into the ligand-receptor interactions [85].

## 5. Conclusions and Future Research

Since the three-dimensional crystal structure of NTCP is not available, protein engineering involving site-directed mutagenesis, missense and nonsense mutations and generated chimeric constructs were prerequisites aiming at understanding the structure, topology, and significance of particular protein motifs in regulating NTCP’s activity. Comprehensive mutational analyses included over seventy individual amino acids from the entire human 349 aa-containing NTCP sequence. This accounted for approximately 20% of the whole NTCP protein sequence and resulted in a generation of over hundreds of NTCP mutants, which were further subjected to in vitro and in vivo functional studies (Table 1). The obtained results became a valuable information to decipher key residues that contribute to the bile-acid transport and HBV/HDV receptor activities of NTCP. Consequently, several essential motifs, protein modifications and features have been identified and deeply characterized. These include but are not limited to (i) HBV receptor binding domains (_84_RLKN_87_ and _157_KGIVISLVL_165_), (ii) natural physiological substrates and specie-dependent substrate specificity, (iii) bile acid and Na^2+^-binding cavity, (iv) natural variants of NTCP (S267F and R252H), (v) oligomerization state, (vi) posttranslational modifications (N-glycosylation and PKC-dependent phosphorylation, (vii) protein trafficking (c-terminal membrane localization sequence, PKC and clathrin-dependent endocytosis) and (viii) novel entry inhibitors for HBV/HDV infection. As result of those protein engineering studies, the considerable progress has been made to understand the molecular structure, biology of multitasking NTCP molecule and underlying mechanisms of its activities. Nevertheless, certain limitations of experimental approaches such as the influence of missense mutations on protein stability, aggregation and folding, often resulting in “loss-of-function” of the genetically modified protein, leads to misleading conclusions, which shall not unquestioningly be considered.

There are still many unresolved aspects related to NTCP structural biology, which need to be addressed soon. First, the 3-D NTCP structure as well mechanistic insights into conformational changes after ligand (physiological substrate or virus) binding must be determined. Second, NTCP membrane topology under conditions mimicking a membrane-like environment needs to be assessed. This can be achieved using, for instance, a nanodisc technique engaging membrane scaffold proteins combined with a single-particle electron cryo-EM, which represents the innovative technology for the structural determination of integral membrane proteins and protein complexes. Third, using the above-mentioned technique, it is indispensable to decipher the underlying mechanism of NTCP and HBV interaction and crosstalk between host-derived proteins and invading pathogens. This includes a confirmation of identified virus-binding motifs as well as the recognition of novel NTCP regions required for virus docking and internalization upon infection. Moreover, given an increasing number of NTCP-interacting partners and the NTCP-self oligomerization that have recently been identified as key players during HBV infection [19,63,71,76], it is fundamental to evaluate their detailed contribution to structural changes of the NTCP/ligand complex. To functionally address all aforementioned NTCP-related topics, the purification of NTCP protein under native conditions is desperately needed. Due to the lack of an appropriate structure, approaches for the development of HBV/HDV entry inhibitors are restricted to the ligand-based approach. Thus, determination of the 3D structure of the virus/NTCP complex would enable an additional structure-based approach that would be much more specific and promising. This would significantly help to design novel pharmacological strategies aiming at blocking NTCP-driven HBV internalization into human hepatocytes.

## Data Availability

Not applicable.
